# EXAMPLES OF ENGAGING HARD-TO-REACH POPULATIONS IN CLINICAL RESEARCH

**DOI:** 10.1093/geroni/igad104.1307

**Published:** 2023-12-21

**Authors:** Evan Plys, Melissa Gerald

## Abstract

Individuals, communities, and settings that are systematically and historically under-represented in research are sometimes referred to as “hard-to-reach” populations. Investigators often face additional challenges to engaging hard-to-reach populations, as research commonly occurs in settings or situations where clinical science has not historically taken place. The perspectives of hard-to-reach participants are vital for moving toward health equity. Yet, investigators must be equipped with specific strategies and tools to conduct high-quality clinical research with hard-to-reach populations. In this symposium, a diverse panel of speakers will present examples of research with various hard-to-reach patient populations and settings. The first discusses research targeting socioeconomically disadvantaged short-stay skilled nursing residents and their care-partners. This presentation highlights challenges and solutions for partnership formation, budgeting, recruitment, and engaging community advisors in skilled nursing facilities. The second centers on research with Hispanic families caring for an elder living with dementia. This presentation highlights challenges and solutions for recruitment as well as language translation and cultural adaptation of study materials. The third discusses research with incarcerated older adults and those receiving treatment from outpatient substance use disorder clinics. This presentation highlights challenges and solutions for recruitment, assessment, and adapting protocols in these settings. The final discusses research with adults receiving Hospice care for advanced cancer. This presentation highlights practical, ethical, and cultural considerations for engaging participants in their final months of life. We conclude with a broader discussion of approaching clinical research with hard-to-reach populations, with the ultimate goal of inspiring investigators to enhance the reach of their science.

